# Ketamine-assisted psychotherapy in adolescents with multiple psychiatric diagnoses

**DOI:** 10.3389/fpsyt.2023.1141988

**Published:** 2023-03-30

**Authors:** Philip E. Wolfson, Julane Andries, Daniel Ahlers, Melissa Whippo

**Affiliations:** ^1^The Center for Transformational Psychotherapy, San Anselmo, CA, United States; ^2^Ketamine Research Foundation, San Anselmo, CA, United States

**Keywords:** adolescent psychiatry, major depressive disorder, bipolar disorder, ketamine-assisted psychotherapy, ketamine, psychedelic psychotherapy, eating disorders, adolescent mental health crisis

## Abstract

Ketamine-assisted psychotherapy is a promising new treatment for a variety of mental disorders of adolescence. There is currently an adolescent mental health crisis, with a high prevalence of disorders, diagnostic complexity, and many adolescents failing to respond to conventional treatments. While there is strong evidence for the use of ketamine in adults for a variety of treatment-refractory mental illnesses, research in adolescents is in its early stages. Ketamine-assisted psychotherapy (KAP) has been described in adults with promising results and here we present the first published cases of the use of KAP in adolescents. The four cases include adolescents aged 14–19 at the initiation of treatment, each with a variety of comorbid diagnoses including treatment-resistant depression, bipolar disorder, eating disorders, anxiety, panic, and trauma-related symptoms. They each initially received sublingual ketamine, followed by sessions with intramuscular ketamine. Their courses varied, but each had symptomatic and functional improvements, and the treatment was well-tolerated. Subjective patient reports are included. Rapid resolution of symptomatology and suffering often occurs within months as the result of the application of KAP to adolescent psychiatric care but is not inevitable. Family involvement in the treatment process appears to be essential to success. The development of this modality may have a singularly positive impact that will expand the psychiatric toolbox and its healing potency.

## Introduction

There is a recognized and alarming crisis in adolescent mental health worldwide (1), both preceding the COVID-19 pandemic (2), and certainly in the midst of it, with evidence suggesting a doubling of the prevalence of depression and anxiety (3). From May 2020 to March 2021, the rate of emergency department visits for suspected suicide attempts increased precipitously for adolescents aged 12–17 compared to the corresponding period in 2019, particularly among female subjects (4).

Mood disorders in adolescents are common, often with multiple comorbidities (5), and are associated with poorer outcomes than those with later onset (6). These disorders are associated with an elevated risk of suicide (7, 8), substantial disability, and societal burden (9). Longitudinal studies demonstrate significant associations between childhood mental disorders and children's health as well as with their caregivers. Prospective and long-term studies of sub-threshold symptomatic adolescents and diagnosed adolescents indicate significant progression into adulthood of mental disorders, emphasizing the need for treatment, access to care, and the impact of social and economic factors (10–12). Of great concern is that young people are prescribed a panoply of medications for mental health concerns (13), and yet, many do not achieve remission or do not respond to the current evidence-based or FDA-approved treatments (14, 15).

Ketamine has been shown to yield rapid and clinically significant positive effects in adult treatment-resistant mood disorders (16–18). One of the more robust outcomes of treatment with ketamine is a reduction in suicidal ideation (19). A recent large double-blind randomized controlled trial demonstrated that 63% of adults receiving ketamine achieved sustained remission of suicidality (*n* = 17) using a single dose of 0.5 mg/kg of IV ketamine and assessment over 2 weeks (20).

Given the success of ketamine in adults and concerns for the state of the mental health of youth, there is a growing interest in its application to adolescents. A systematic review summarized the nascent research on ketamine for adolescent treatment-resistant mood disorders (21). They concluded that ketamine was shown in adolescents to improve depressive symptoms, decrease acute suicidality, and reduce mood lability, however, a number of subjects did not have significant responses to treatment. This review identified one study concerning bipolar disorder (22)—a retrospective chart review of 12 youth receiving insufflated (30–120 mg) racemic ketamine. The remaining articles were reports of IV ketamine (0.5 mg/kg infused over 40 min) for adolescent treatment-resistant depression. One open-label trial (*n* = 13) (23) and two case reports were presented (24, 25). A supplemental case report of a depressed adolescent who received a continuous IV ketamine infusion over 5 days for chronic pain, but whose depression improved along with the pain, is also mentioned (26). A single intravenous or subcutaneous administration of esketamine to 10 adolescents with a mean age of 15.5 years and a variety of diagnoses and psychiatric and medical comorbidities resulted in a 24-h reduction in depressive symptoms and suicidal ideation (27). A single dose of IV ketamine in adolescents with depression was found to be well-tolerated acutely and with significant short-term (2-week) efficacy in reducing depressive symptoms compared to midazolam as an active placebo (28). Wink et al. (29) provide preliminary evidence for brain correlates of clinical change based on neural flexibility utilizing fMRI. They hypothesize this may underlie symptom relief in adolescents with TRD following six infusions of ketamine.

Autism spectrum disorder (ASD) and ketamine are in early exploration with a report of safety and tolerability of intranasal ketamine in 14–29-year-olds (30) and a single patient report of a dramatic brief remission of the core symptoms of autism (31).

The DiVincenzo meta-analysis of adults and adolescents (19) reports the safety and tolerability of ketamine for adolescents and the improved success of higher doses than the standard 0.5 mg/kg with no substantial adverse effects, nausea being the most common and similar in frequency to adults. They cite a “rapid and robust antidepressant response in adolescents” with “better antidepressant outcomes for individuals who received longer treatment courses and higher doses,” which applies to both adolescents and adults. It is worth noting that the review appears to have included the same number of adolescents−33– as in the Kim paper cited earlier (21).

## Basic pharmacology of ketamine

Ketamine presents in two enantiomers: the S(+) and the R (–) configurations and as a hydrochloride salt. Historically and in general use for off-label psychiatric indications, as well as for anesthesia and analgesia, is an equimolar racemic mixture of the two enantiomers. Although racemic ketamine has the broadest worldwide use, S(+)-ketamine is available in some European countries and came to market as a commercial patented nasal preparation for psychiatric use in 2019.

Ketamine's most probable mechanism of action is as an N-methyl-D-aspartic acid (NMDA) glutamate receptor antagonist. It may well be that its principal site of action is at a specific NMDA receptor that has a dual capacity as a locus for both antidepressant and dissociative attributes. When NMDA receptors on gamma-aminobutyric acid (GABA)-ergic neurons are antagonized, downstream glutamatergic neurons are disinhibited. This increased glutamatergic activity impacts neural signaling, synaptic plasticity, and connectivity. It is posited based on animal models that ketamine-induced synaptic potentiation and proliferation may play a key role in eliciting antidepressant effects. Ketamine also impacts other neurotransmitter systems, affecting cholinergic, opioidergic, monoaminergic, and GABAergic functions [see Wallach and Brandt for a comprehensive review (31)].

Multiple routes of administration are utilized by practitioners treating depression and other psychiatric conditions, each with its own unique pharmacokinetics, including intravenous, intramuscular, intranasal, sublingual, subcutaneous, epidural, anal, and oral delivery.

## Safety of ketamine

McCann and Soriano reviewed the preclinical evidence of the potential for ketamine and other anesthetic agents to induce neurotoxicity in the developing brain (32). While the neurotoxicity of ketamine has been demonstrated in animal models, its clinical relevance is questionable. Examples of studies showing both neurodegeneration and neuroprotection in the developmental period are cited (33, 34). They note that the relevant factors include (1) susceptible developmental age, (2) high dose of the anesthetic, and (3) long duration of exposure. When neurotoxicity is demonstrated, doses are much higher and for a much longer duration than in clinical use. For example, Slikker et al. showed that rhesus monkeys in earlier developmental stages [122 days of gestation and 5 post-natal days (PNDs)] given ~10 times the dose needed for sedation in humans and for a period of 24 h appear more sensitive to ketamine-induced neuronal cell death than at 35 PNDs, and a shorter duration of 3 h of ketamine anesthesia did not result in neuronal cell death at five PNDs (34). Soriano goes on to note that a clinical manifestation or phenotype of anesthetic-induced neurodegeneration has not been identified in humans, providing reassurance for the continued use of general anesthesia in children (33).

There are numerous clinical studies of ketamine for anesthesia and procedural sedation in children and adolescents indicating its safety (35–38). In addition, a study of nine children inadvertently given 5–100 times the intended dose of ketamine in the emergency department showed no adverse outcomes, although prolonged sedation occurred in all cases and four experienced brief respiratory depression (39).

Importantly, Lee et al. (40) discuss critical time windows for neurocognitive damage in primates and humans, the latter being particularly susceptible during the developmental window from *in utero* to 3 years, with the possibility of more time due to ongoing synaptic plasticity. There is controversy and ambiguity with respect to this with both ketamine and GABA antagonist anesthetics. The byword is caution in neonates and young children. Animal studies contribute to this complexity with evidence of both neurotoxic and neuroprotective impacts from ketamine (40).

Long-term pediatric use of ketamine on a repeated basis has largely been confined to analgesia with the demonstration of its safety. Long-term continuous infusion for pain has been reviewed with three studies of 4–14 days duration without significant side effects—and at doses higher than our episodic intermittent use in adults (41). As clinical use of subanesthetic ketamine has vastly increased for a variety of psychiatric disorders, so too has the need increased for prospective studies of long-term effects.

Given ketamine's episodic, intermittent, and low-dose usage for psychiatric treatment, we would argue that the effects of continuous mood stabilizers, antipsychotics, stimulants, tranquilizers, and antidepressants present a much greater and unassessed risk to adolescent brain maturation. Furthermore, the negative effects of these on emotion and cognition are well described. It is clear that the risks to the youth of emotional disorders and morbidity—including suicide, self-harm, eating disorders, impulsivity, alcohol, and drug abuse—outweigh the potential risk of ketamine use in a controlled clinical application.

## Ketamine and KAP

There are many articles on the intravenous use of ketamine for treatment without psychotherapy, generally in medical settings administered by anesthesiologists [(42–44)—as examples]. In this context, attendance to the subjective experience of the ketamine patient is generally absent. Receiving a substance that causes small to large dissociative experiences without the ability to process these experiences tends to diminish their value and often leads to confusion and a sense of “where did I go?” and “what just happened to me?”

In contrast, the value of ketamine embedded in a psychotherapy context is becoming more widely established both clinically and in the literature. We have previously described our process of ketamine-assisted psychotherapy (KAP) and published preliminary data suggesting its effectiveness in the adult population (18). The subjective experience of ketamine, or signature, can be characterized as a spacious time-out from the ordinary mind and its obsessions, with an increased capacity to observe and let go of dysregulated thoughts and behavior patterns. Psychotherapy provides support for a full expression of the subjective experience of ketamine within a conducive non-medicalized setting (18, 45–50). We have used this method in over 1,500 patients and many thousands of sessions in the adult population and have found KAP effective in facilitating new modes of being. This may be particularly relevant in the adolescent population, as most large studies have suggested that the combination of psychotherapy and medication is superior to either treatment on their own for anxiety disorders and depressive disorders. Supporting this furthermore, a recent study of parent perspectives on ketamine suggested that although much of the research to date has been on IV ketamine, parents were more open to less invasive modes of administration, and the authors speculated on the potential for ketamine-assisted psychotherapy to help address some of the understandable parental concerns (50). Our work with adolescents is always in the context of family therapy, including parents and situating adolescents in their homes, schools, and social matrixes.

We work dyadically (i.e., a medical doctor and therapist for each patient) with all adolescents as is customary in working with alternative medicines, such as MDMA (51), and in our practice with ketamine-assisted psychotherapy in adults. KAP sessions are long, generally lasting 3 h or more, and therapist fatigue is a factor mitigated by involving multiple clinicians. The nature of the transference is altered by working dyadically and may offer an emotionally corrective experience of a healthy couple's interaction. Projections based on mother and father experience offer the opportunity for exploration, awareness, and change with processing between therapists and patients.

Generally, adolescents and their families seek our ketamine program after failure with conventional treatment that may include multiple antidepressants, antipsychotics, and mood stabilizing agents, and for a wide variety of clinical presentations. Parents come to us with feelings of confusion, frustration, helplessness, and conflict exacerbated by fear for their child and their own sense of inadequacy. Often, they are in eating disorder programs and have had multiple therapists. While treatment-resistant depression was the major diagnostic category for which intravenous ketamine treatment was based in the adult population (52), ketamine in its general adult application has come to have far greater diagnostic indications than TRD and the literature is voluminous in this regard [see (18, 52, 53)].

## Methods

Intake and assessment measures are standardized across patients.^1^ We administer a detailed questionnaire along with measures at intake to assess for childhood adverse experiences, resilience, depression, anxiety, and PTSD. We assess changes at each session with repeated measures and our own change of state form, as well as for the effects and presence of mystical experience and depth of ego-dissolution during ketamine experiences. We have our own charting at each session for the practitioner assessment of changes in anxiety, depression, PTSD, personality rigidity, sensitivity to ketamine's effects related to dosage, personality rigidity, changes in diagnosis and medication, psychodynamics, social and family system changes and stresses, school experience, friendship statuses, and the patient's view of their experience. We have developed our own Redcap Vanderbilt digital format for recording comprehensive data for each patient, changes in their status, and termination. Diagnosis is made by review of intake materials and medical records, through initial clinical assessment, as well as through consultation with outside treating practitioners, family members, and our weekly case conferences.

Written informed consent is reviewed with parents and adolescents and signed by all involved after satisfying any concerns and questions. The possibility of withdrawal from treatment is present at any time, before, or during treatment, up to the period of administration of the ketamine. Our informed consent is detailed as to effects and potential adverse effects and the rationale for ketamine's use. This is repeated in verbal interaction.

Once appropriateness for treatment has been established, we administer ketamine using two routes of administration: sublingual (SL) or intramuscular (IM) injection. These routes of administration can be administered safely in an office practice where the environment facilitates a comfortable setting for the ketamine experience, in contrast to the often medicalized setting of IV use. Multiple dosing strategies are available for each route of administration and are tailored to patient needs and responses.

We typically initiate treatment with SL administration, using oral dissolving lozenges for dosage assessment and patient familiarity with the effects. Lozenges are held in the mouth for 15 min before spitting or swallowing based on patient preference and increased nausea with the swallowing of larger doses. The SL method provides a slow and gradual onset of effect, enabling close contact between practitioner and patient and minimizing the impact of disorientation as ketamine exerts its effect of moving patients into nonordinary states of consciousness.

If appropriate, the treatment then may include intramuscular (IM) injections. The IM route of administration provides rapid onset of 2–3 min. Generally, the clinician's awareness of sensitivity to ketamine has been established by the sublingual sessions that have preceded the IM use, allowing careful selection of appropriate doses. Up to three injections are typically used for 40 min, with the aim of increasing depth or duration by timing to the rapid metabolism of ketamine.

The choice of route of administration is clinically determined and is provided in a carefully tailored manner beginning with a low first dose and stopping at a dose that is effective for ego-dissolution. IM dosing allows for flexibility of the clinical choice of depth of experience—from mild alteration of consciousness to full ego-dissolution and an internalized experience separated from external sensory input. This state is valued for ketamine's effects for a variety of reasons. Primarily, it is a time-out from the symptomatic and obsessional preoccupations that are the expression of the patient's suffering. In the case of an adolescent, it may be felt as a relief from the pressures causing symptomatic behavior, allowing for a reformation of consciousness and attitudes. With therapeutic guidance, this experience opens new possibilities for self-understanding, self-control, and behavior. The achievement of this generally requires repetition of ketamine experiences, bonding with therapists, and understanding and support for the psychosocial culture and its participants. In addition, the ketamine experience itself confirms the adolescent's inherent capacities and imagination, which have become blocked by the parts that are dysregulated and reactive. The ketamine space is interesting, free of usual concerns, and tends to have a neutral to loving and self-appreciating effect. This positive reclamation may well lead to a reduction in self-loathing, despair, suicidality, and reactivity, opening the door to healing.

A decision to move from SL to IM, or to combine both, is based on successful experiences at the lower doses with minimal side effects and the decision with family and adolescents for a deeper experience based on the perception that there will be a greater impact on symptoms. Patients may have a full therapeutic response with only SL experiences.

There is no accurate method to relate sublingual (or intra-nasal) dosage to intramuscular dosage—estimated at 95%. As with the esketamine intranasal data (54) which indicates a broad range of absorption, the SL method's range of absorption depends on local oral conditions, hydration, the nature, and time for the dissolution of the oral format, and especially the duration of holding the saliva that is the ketamine carrier in the oral cavity. No study has formalized the latter or actually focused on it. In our clinic, we have optimized holding the saliva for 15 min. Our rapid dissolving tablets dissolve in 1–2 min. One product supported study found a median absorption of 29% of the administered dose (55), with another 17–27% (56). Our estimate is of a 15–30% absorption over the duration of exposure. With swallowing, there is a secondary minor absorption from the small intestine with various estimates clustering around 10–15% of swallowed ketamine. As we begin our assessment of ketamine's effect with a low-dose dosage finding procedure, we later can make estimates of the sensitivity to the more rapidly absorbed ketamine administered intramuscularly.

Early in our learning about ketamine, other clinicians noted that several of their patients had abused the prescribed intranasal (IN) ketamine and had become dependent on it. We came to understand that the abuse potential of the IN route was an issue and we prefer the SL method as it is difficult to fill one's mouth with an excessive number of lozenges in contrast to the ease of overuse of the IN administration.

In this regard, the administration of SL lozenges at home is a mainstay of our KAP work. We are aware that at-home ketamine lozenge use has become a major commercial enterprise and is open to critical scrutiny in terms of the thoroughness of the programs that distribute the lozenges, controls on use and dosage, and a lack of psychiatric support. Nonetheless, its use in this context has been recently studied with reports of safety in widespread use (57, 58); however, these articles have come under methodological criticism (59).

Our clinical provision of ketamine for at-home sessions is quite different in being far more supervised and embedded in our face-to-face ongoing KAP program. For our adolescents, this means that parents are in possession of a limited quantity of the lozenges we prescribe, with no opportunity for automatic refilling. Adolescents must request lozenges from parents. Parents are supervising sessions under the direction of our practitioners. We are available for consultation and the handling of potential crises or safety issues (which have not occurred to date, with at-home use under regular review). The advantages of at-home use are multiple and include an increase in the frequency of sessions and decreased cost, facilitating an increase in the success of our program due to the availability of treatment. At-home work with adults enabled us to provide and continue treatment during the COVID-19 pandemic when in-office sessions were impossible. With careful monitoring of prescriptions and supervising timeframes for use, we have had no safety issues, misuse, or diversion in thousands of at-home sessions in our adult patient population.

At-home strategies for ketamine use for adolescents have three major programs. The first entails supportive sessions at doses of 100–300 mg SL which provide a degree of the time-out experience described earlier. The frequency of such sessions is tailored to the specific therapeutic plan, with more frequent dosing in the early phase of treatment—up to two times per week. These sessions support in-office work and improve outcomes. The second strategy is designed to reduce acute anxiety and consists of a 50–100 mg SL experience which can be repeated at this low dose multiple times per week to reduce the anxiety that results in activated dysregulated behavior. It can be requested by the adolescent or suggested by the parent(s). We limit the number of such sessions and supervise their application. The third strategy is designed to interrupt the impulse to self-harm or indulge in eating-disordered behavior. When the impulse begins to be felt by the adolescent, they have been taught to request a 50–100 mg lozenge which allays the anxiety that attends the potential behavior. This application facilitates a consciousness about the feelings that are about to motivate the behavior and may lead to self-control without ketamine.

This article describes our work with four adolescents with severe and complex presentations and is to our knowledge the first to describe the effects of ketamine-assisted psychotherapy in adolescents. The adolescents discussed as subjects herein were self-referred to our clinic by parents and therapists in our community knowing of our work. No recruitment was made, and treatment was entirely voluntary and conducted with informed consent (IC) that was discussed with parents and adolescents at length. Questions and concerns were addressed to their satisfaction to proceed with KAP treatment. Withdrawal from treatment was always possible as per the IC. No advertising was conducted. The case examples are drawn from the clinical experience of subjects and parents, and when present, in consultation with treating physicians and therapists.

## Informed consent

The following proper names are pseudonyms, and any details of identity are obscured for confidentiality. The patients and parents provided their written informed consent to participate in their treatment and for anonymous inclusion of their experiences in this article.

## Case examples

### Patient Andy—Case report

Keywords: major depression, suicidality, anorexia nervosa, parental conflict, verbally abusive father

Abstract: Abstract: An adolescent with complex self-destructive behavior, actively suicidal, highly reactive to father's verbal abuse and rages, and mother's passivity, who has been successfully treated with ketamine-assisted psychotherapy with relatively rapid resolution of dysregulated behavior and improvement in family stressors with family therapy.

Andy began with us at age 14, in the eighth grade, at the edge of anorexia at 120 lbs. on a 5'9' frame and after two antidepressant failures (treatment-resistant depression by definition). Diagnoses on admission were major depression (F33.2), suicidal ideation (F45), intentional self-harm by a sharp object (X78), and anorexia nervosa (R63). These diagnoses were continuations from the prior treatment and hospitalizations and validated at intake and in the course of treatment.

There were no prior significant medical, birth, developmental, or genetic histories. The family structure is of importance to the dynamics of Andy's struggle. The father is of Latino background, grew up at the edge of the barrio in New York City, and managed to attend prestigious college and law schools by dint of severe self-discipline. The imposition of this in the family was a major source of conflict. In contrast, Andy's mother was of Irish background and much more laissez-faire. Prior treatment focused solely on the eating disorder and hospitalizations for suicidality. A short stint of psychotherapy was abandoned as ineffectual.

#### Mental status evaluation

Andy appeared agitated and hyperverbal with anger, volubility, and psychomotor agitation. Grooming and dress were appropriate. There were healing cuts on his arms and keloiding of prior lacerations on his legs. He was extremely thin and spoke of restricting food intake. He expressed anger and fear of his very successful father. Andy is highly intelligent, though with a great deal of self-consciousness, denigration, and fear for his abilities. He demonstrated self-awareness and a capacity for introspection. He also was candid about his potential for further cutting and suicidal behavior which made intervention urgent. There was no evidence of hallucinations or delusions, but grandiosity and dramatic expressions were part of his presentation.

Parents were interviewed separately, together, and with Andy present. A 2 year younger daughter was of concern as the parents indicated she was depressed and expressing suicidal thoughts and feelings of worthlessness. She was to start therapy with her own therapist.

On Intake: ACE 2; Resilience 11; BDI 47; HAM-A 35; PCL 59.

At the time of this report (12 months): BDI 2; HAM-A 2; PCL 25.

Andy attributes his depression and subsequent anorexia to sixth grade and his father's dominance, as well as verbal abuse. He made three suicide attempts in 2021—one overdose with 20 Advil and 500 ibuprofen. There were two hanging attempts. Serious ongoing cutting episodes were occurring with deep wounds to thighs and arms and subsequent keloiding. Andy's compulsion to cut and control his food intake was voiced openly. Parental controls were ineffective, and splitting was both active and passive.

Andy had no history of substance abuse. Medication consisted of a recent prescription of citalopram 20 mg which was discontinued as ineffective after our evaluation. There was no history of alcohol or substance use or abuse. Except for the ketamine, no other medications were administered during the course of treatment.

Working as a dyad, our focus was immediately on the family and Andy's expressions as the identified patient, reflecting his hopelessness, rage, and anxiety. The parents were in a near-constant state of quarreling and yelling with violent verbal abuse. Underneath his resistance to his father's bullying was a terrified sense of not living up to his father's significant successes.

We initiated family, couple, and individual therapy. The father initially begrudgingly took responsibility for his behavior. As he came to a clearer understanding of his own rage and feelings of disrespect, he softened in his relationships. Over time the couple's open friction reduced. Therapy was not particularly successful in assisting the mother in reducing her provocative rebelliousness, this expressed as a compulsion to be late to virtually every scheduled event, which both Andy and his father noted as a source of tension. The success of the focus on the conflictual atmosphere in the home was of tremendous import to the work with Andy as it validated his sense of being harmed by the conflict and reacting to it with his own despair and acting out behavior.

With Andy, KAP therapy focused on increasing his autonomy and self-worth and moving to a position of recognizing the cost to himself and the futility of his self-destruction. His impulses to cut and abstain from eating continued but with less pressure. At the time of writing this, we are at 1 year's treatment without a cutting episode. This has become a source of pride for Andy. There are still occasional thoughts of self-harm but none of suicide. Andy has successfully matriculated into a prestigious high school.

#### Course

Ketamine's role in the transformation of this family in only several months' time has been profound. Ketamine sessions were initiated with our SL method, commencing at a low dose (100 mg in two tranches based on Andy's response) and building comfort with the inevitable disorientation of ketamine's effects. Andy's intelligence and his increasing self-awareness of his pain, impulses, and the causes of his reactivity at the moment grew rapidly as the work progressed. Ketamine sessions moved to higher doses providing more complete ego-dissolution and relief from the tensions of his ordinary life and obsessions. Andy's intention—always elicited before ketamine administration was: “I would like to understand my depression and be done with it.” The SL dose at the second session was increased by 50 mg. Andy moved across the couch swimming like a fish, and overtly happy. At integration, he expressed: “I can be happy if I wanna be in this ketamine space” and, “How can I explain to my dad that I am a fish?” He called his girlfriend and told her “I need to regain cognitive functions.”

Given his safe and positive response to ketamine, with parental permission, we initiated a third KAP session with a 50 mg lozenge easing him 20 min later into a 50 mg intramuscular experience. This intentional increase in ketamine was and is designed to induce a state of deeper relief from the pressures and stress of usual consciousness and with a resultant improvement in perspective and reduction of reactive, symptomatic thought and behavior. Andy entered a deeper state than previously without difficulty. At integration, he stated: “I never realized self-harm wasn't going to help. Why is the urge to self-harm there? I never knew when to stop and draw the line to self-harm. My essence is to be kind to myself.”

We supported this intention and realization by providing the parents with lozenges to be provided to Andy at a low dose, either at his request to prevent self-harm or by their perception of an increase in tension and stress, raising the possibility of self-harm. The interruption of rising anxiety and the impulse attached to relieve that anxiety by a self-destructive action utilizing a low-dose at-home ketamine experience has become a treatment modality we have incorporated into our therapeutic work. It is entirely under parental control and is described at length above. Andy used ketamine at home on occasion in the low-dose format to reduce the anxiety that would have led to self-harm.

We have completed 13 in-office sessions over 12 months. Our KAP session dose has increased to 90 mg IM with an increase in the depth of each journey that is welcomed by Andy. He values the relief from his stress and the experience of the ketamine journey. We are entering a maintenance phase, with sessions occurring based on struggles and risk for decompensation—which has not occurred. Integration sessions without ketamine, telephone and email support, and consultation with parents continue. Ketamine use at home has always been carefully monitored and has become less frequent. Recently the mother requested a ketamine session and had a remarkable experience benefitting her ability to express herself with a reduction of fear and inhibition. This is having a positive effect on the couple.

The essence of the ketamine phenomenological signature is a time-out enabling freedom from usual concerns and the opportunity for a new view of self and context (1, 18). This provided Andy with the emotional space for him to constitute a sense of personal value separated from the family conflicts, thus resulting in the enhancement of friendships and activities outside the home. Andy has a remarkable capacity to feel and express his inner life. This increased with his sessions as did his sense of imagination and pleasure in his own creative mind. Our 3-h plus sessions included ongoing family work in the post-ketamine period after Andy's reintegration. His transference to us as good parents, the understanding and acceptance that facilitates self-regulation, and a deeper sense of trust in his own judgment and behavior. With Andy's rapid recovery, that sense of trust in us was extended to us from the parents as well, and we enabled a shift in focus to them and an enhancement of mutual kindness and respect. A profound sense of relief was the result. Therapy with the parents continues and our work has had a positive impact indirectly on the younger daughter who was becoming symptomatic.

There were no significant adverse effects. Tapering of ketamine's use has been without withdrawal or cravings.

Ketamine's effect as an anxiolytic and as an interruption of intent has proven to be an effective intervention in this aspect of working with all manner of impulses, including binging, cutting, suicidal ideation and contemplation, and other potential methods for self-harm. We view the rapidity of effect to be of great therapeutic potential.

#### Patient Andy's perspective comments

After having multiple in-office ketamine sessions and occasionally taking the at home[sic] ketamine tablets, I have noticed a major reduction (to the point of non-existent) in suicidal thoughts, tendencies, and urges. I have noticed a major reduction (to the point of non-existent) in self harm[sic] thoughts, tendencies, and urges. A major reduction in depressive feelings and depressive mood. Overall better wellbeing feeling.

There were no difficulties in[sic] the medicine apart from nausea on 2[sic] separate occasions.

I did not find difficulty in the journey experience but rather found it to be enjoyable and I found being able to navigate through it to be quite helpful and with relative ease.

I would definitely do this treatment again. I would also highly recommend this treatment to anyone who is troubled by depression, suicide, or self-harm aspects in their life.

I had absolutely no trouble reducing or stopping ketamine use.

### Patient Bianca—Case report

keywords: PTSD, anorexia nervosa, panic disorder, divorce, parent alienation syndrome.

Abstract: A highly intelligent, regressed teenager caught between parents who had divorced when she was 1 year old and continued their relationship in a constant struggle over custody, splitting Bianca into a loyalty struggle. Bianca has an extensive history or physical trauma, potential sexual molestation, PTSD from being at a shooter incident, suppression of her individuation and desires for growth and expression, and a great deal of resultant frustration and anger. Her eating disorder commenced as a deliberate attempt to have her parents, particularly the mother, pay attention to her and her determination to have her presence and desires taken seriously. Family therapy with the intention of reducing the parental conflict and unity around Bianca's needs was blocked by the mother who made efforts to stop our work throughout its course. KAP served Bianca over time to verbalize her feelings and needs, to exert her own will through her self-determination, and to gradually leave behind the impactfulness of the anorexia which had taken on a life of its own.

#### Bianca

Bianca presented to our program as a 14-year-old girl with prior treatment for precocious puberty between ages 7 and 10, a significant history of trauma, with related distrust, hypervigilance, panic symptoms, depression, and an eating disorder. Her diagnoses were PTSD (F43.10), major depression (F33.2), anorexia nervosa (R63), and panic disorder (f41.0). These diagnoses were made in prior treatment and validated by us at Intake and subsequently. There was a history of multiple physical accidents, and possible sexual and physical molestation by her stepfather. Bianca had reported this at age 4 and a Child Protective Service inquiry was inconclusive. Later, she would be open about her fear of her mother's second husband and avoided contact with him, not feeling protected by the mother who had not taken any action on her behalf to protect her from her husband. She had been present at an active shooter incident with a resultant stampede at an amusement park at age 12 that left her with circumstantially related panic attacks.

At intake, she was prescribed Sertraline 50 mg and melatonin for sleep. Prior medications included only sertraline. There was no history of self-damaging behavior; alcohol or substance use or abuse, her sertraline was increased to 75 mg by her eating disorder MD early in our treatment. She is currently in the course of reducing her sertraline dosage. There has been no substance abuse whatsoever during her treatment.

Mental status evaluation at intake indicated an appropriately groomed extremely thin young woman with significant facial acne. She was short adding to the sense of her diminutiveness. Taciturn, withdrawn, with deeply depressed affect and avoidance of eye contact, she huddled in a corner of our couch. Questions were answered minimally, and she offered nothing voluntarily. When her parents left the room and she was alone with the female and male dyadic therapist pair, she came a bit more alive and there was superficial contact and greater responsivity. There was no evidence of a thought disorder, hallucinations, or delusions. Suicidal ideation was acknowledged without current interest in an attempt. Of above-average intelligence, it was only over time that we were able to understand the degree of her intuitive and psychological depth. We rapidly became aware of her strong will, mostly exerted at this time about eating and struggling with the stringency of the eating disorder program which mandated every morsel and type of food she was to consume—a constant struggle. Bianca was in weekly therapy with a female therapist of her mother's choosing for whom she had some fondness. The precocious puberty at 7 had been difficult for her and treatment had continued until age 10.

Her PTSD Checklist (PCL) score at intake was 51, suggesting a high level of clinically significant PTSD symptomatology. It is now 39. BDI has gone from 42 to 14.5; Ham-A from 31 to 15—these last results from our last session, the 38th.

The mother is of Chinese background. The father is of Jewish background. There is no significant mental illness or physical or genetic disorders on either side.

At intake, she was in weekly therapy and in a regimented eating disorder treatment program to which she had become dependent, not eating on her own without direction. Her parents were both present at intake, though her father initiated treatment after being referred to us. Her father was present at most of the sessions, while her mother was present at two sessions.

Her parents divorced when Bianca was 1 year old, and this ongoing protracted contentious divorce, in which Bianca felt that she was in the middle, was a central stressor in her life and presentation. She reported feeling coerced by her mother to choose her side, with threats to the father's custody, including it having been blocked for a time. Contention over the father's ability to act on her behalf and threats to reduce contact was and continues to be major theme throughout her childhood. This splitting and contention continue to the present with Bianca, now 15, with an increasing ability to decide on her life path and choices autonomously. We considered as per Gardner (60) a degree of parental alienation syndrome (PAS) at work.

The unfortunate acrimony between the parents enters directly into our therapy with Bianca. This consists of her father, who throughout her life she experiences mostly as the good parent and her advocate, and the mother as the controlling, negative force. Our efforts to build an alliance with the mother and build unity for the child's sake were rebuffed and the mother has attempted to stop the KAP treatment despite its success and with Bianca's avowed desire to continue with the treatment. This has finally ceased as Bianca has exerted her own determination to continue.

Historically, there were multiple traumas under her mother's care—who remarried when Bianca was 2. These include being bitten by a dog at age 2 at daycare with punishment for wetting her pants—this leading to a life-long fear of dogs; remarkably she reported to her physicians about inappropriate sexual behavior by the stepfather at ages 2, 3, and 4 with an inconclusive report by Child Protective Services—no effective action having been taken to protect her; a concussion, and lack of appropriate response by the mother at age 10; fractures from falling off a bike at age 10, with no examination until the father took her to the emergency department; refusals by the mother to let Bianca attend the school of her choice (where now 3 years later she is happily in attendance); blocking of her successful acting at age 12 by mother; a near continuous battle in the court over custody and its conditions, the mother now attempting to block Bianca's successful and chosen treatment with ketamine.

The event that triggered Bianca into more recent panic attacks was an active shooter event at an amusement park, and her getting lost in the resulting stampede. The school counselor recommended counseling and the mother refused. At age 13, Bianca took direct action by restricting food intake and was outspoken about this being a deliberate attempt on her part to have her health and mental health issues taken seriously and to not be blocked from her choice of schools and acting. She loses 15% of her body weight, stops drinking, is hospitalized, then enters a partial hospital program; then spends over 2 months in a residential eating disorder program; articulates suicidal plans, and ends up in an inpatient unit for a week.

#### Course

While shy, childlike, and verbally reticent as we began with her, the first session with a single 100 mg lozenge was both well-tolerated and experienced positively as a relief from her usual concerns. Sensitive to ketamine, her experience was significantly dissociative, and she eased into a deep trance state with diminished anxiety and depression as she reintegrated. On the follow-up assessment for the second session, there were very significant reductions in BDI and HAM-A scores. We conducted two more lozenge sessions increasing the dose by 50 mg at each to 200 mg in order to increase the depth of her experience and relief from stress.

Bianca had vivid, imaginative altered states that she enjoyed, feeling a sense of the quality of her imagination and the excitement of her experiences. Bonding with us as therapists enabled early and gentle discussion about family relationships and confidence in our confidentiality and our being for her, with a significant increase in self-disclosure of feelings. Assisting her in building eating autonomy was blocked by her own clarity about wanting the monitoring, nutritional format, and coaching to continue, this having been ingrained as proof of concern for her. Conflicts with her parents in their separate and distant locations would result in regression around food compliance and isolation with depressive and angry moods. This has attenuated.

Given her positive experience with the ketamine lozenges, with her enthusiastic agreement following discussion, we began a course of intramuscular sessions (50 mg) supported by at-home lozenge sessions two times a week with full parental supervision and presence—predominantly the father. This rapidly provided a sense of her own agency and ability to handle the experience with an increasing sense of autonomy. She began attending the school of her choice and after initial nervousness about acceptance, she made friends and has thrived. She resumed her acting and singing. In therapy, she spoke openly about her fear of her mother's irritability, threats to her school about taking her out of it, and bringing her back to the mother's location. Her school choice (an arts and acting focused high school) has unbalanced the custody arrangement as the school's location required living with her father. Her motivation to attend this particular school had occurred before beginning with our treatment, and it was a healthy indication of her beginning moves toward individuation. We discussed the difficulty of her balancing act and its emotional toll. She had become engaged in the therapeutic process. Thoughts of suicide had disappeared early in our treatment. Engagement in living was proceeding.

We reduced in-office session frequency to once per month with our constant availability clear to her. Changes in her dependence on the eating program were occurring and the frequency of meetings with her eating disorder physician decreased. There was a discussion about reducing her antidepressant, initiated by the eating disorder physician and Bianca. We cautioned against it. The program was working. After 5 months, she was eating lunch with her classmates and becoming self-actualizing in her food consumption. Sensitivity to slights by classmates or perceived inadequacies academically caused brief emotional setbacks with reasonably rapid recovery times and processing of these with her father and us. Our ketamine regimen had morphed into two consecutive injections thereby prolonging her sessions—a total of 60 mg—maximizing her time in the journey from 40 min to about 50 min. Ketamine tends to be metabolized at the same rate per person independently of dosage.

Sessions in-office are occurring now every 4–6 weeks and Bianca is slowly reducing the frequency of at-home sessions to once per week. We are all in agreement this is not a long-term process but does include maintenance sessions in future as would be indicated by her needs and her continued psychotherapy with us. This has become a fundamental relationship based on the experience of unwavering trust and support for her autonomy, maturation, and collaboration. Her commitment to her life is sound and strong and her actions reflect this. She will have to manage her balance between her parents but now has the strength to make decisions that further her needs. There will be more work on trauma, pain, and her sense of justice/injustice ahead—no doubt. She is eating entirely on her own and has concluded with her MD and has stopped sessions with her outside therapist who appeared to Bianca and to us in our contacts with her to have taken sides unfortunately with the mother against the father.

This growth and maturation have happened for the most part in less than a year's time with consolidation now over 16 months of sessions. It feels a bit miraculous to us as therapists. Our relationship is with one parent and is strong. Though we made strenuous efforts, we were not been given the opportunity to work with parents as a unity to foster cooperation in the care of their child.

We have had the profound experience of participating in this adolescent's blooming into life. We do not believe that ketamine treatment on its own could have accomplished this. Ketamine combined with adolescent family psychotherapy has. As therapists, we are all too aware that complex and painful divorces are all too common and children suffer in their midst. Unfortunately, the pain and effects continue too often into adulthood.

The role of medicine in this modality is complex. It has engendered a sense of Bianca's capacities and a mind free of pain and struggle—that this is, indeed, a possibility, an actuality in fact. Embedded in the therapy, it has fostered a sense of safety and trust in us that has extended beyond to father and friends. It has acted to reduce depression, anxiety, rumination, and self-harm. It has antidoted hopelessness and despair. It has strengthened trust in her own judgments and lessened reactivity. It has given a sense of self-regard, self-determination, and allowed for a passion for life to emerge.

#### Patient Bianca's perspective comments

I've[sic] experienced [sic]better mood, less anxiety, [sic]more contentment.

Sometimes it has affected my sleep, making it harder to sleep directly after usage, some headaches[sic] and nausea.

Some of my difficulty with journeys has been repetitive imagery. Sometimes when taking oral ketamine, I do not experience images at all.

I would recommend this to friends with ongoing symptoms of PTSD, but only for[sic] people who will take it seriously and use it responsibly. Ketamine's nature is different from many other drugs, and it needs to be treated with respect for someone to get healthy results.

There have been periods of time where I have gone a long time without ketamine and not felt any different, there have also been times that the day before I have scheduled ketamine usage, I feel my mood dropping. I haven't[sic] had any trouble lowering my dosage, I think I would be fine if I did. I think if I did stop ketamine usage, my mental health would drop, but if there was a reason, I needed to I would be able to stop just fine.

### Patient Chris—Case example

keywords: anxiety, panic, grief.

Abstract: A 16-year-old boy presented with a history of 6 years of increasing anxiety and panic, with decreasing functional capacity over the prior 2 years. He experienced remission within 5 months of treatment with ketamine-assisted psychotherapy and the support of loving parents. The themes of the treatment included acknowledging and working through grief over the loss of his grandmother and reemerging confidence in social settings.

Chris is a 16-year-old boy who presented with his parents for help with anxiety and panic attacks. He had been engaged in a psychotherapy process for 2 years with minimal reported benefit and had no prior treatment with psychiatric medication. There was no history of alcohol or substance use. Family history included the mother having experienced depression and suicidal ideation at age 16.

Chris reported that his anxiety began at age 10 for reasons he could not identify and especially worsened over the last 2 years since entering high school. He emphasized a panic attack while attending a dance which he felt “changed” him, with increasing social anxiety that was further compounded by minimal opportunities for social interaction with peers due to the COVID-19 pandemic. He experienced increasing difficulty talking to new people and developed a fear of crowds. His mother reported that when they would go to grocery stores, he would insist on remaining in close proximity to her and refuse to leave the aisle where she was located. He stopped participating on a sports team that he had enjoyed for 3 years prior, feeling paralyzed as he approached the field. He stopped driving, further limiting access to spend time with friends. His multifactorial presentation did not fit any particular DSM diagnosis, so the working diagnosis of an unspecified anxiety disorder (F41.9) was used. On initial assessment, Chris reported the following measures: BDI 19, Ham-A 19, PCL-5 45, Resilience 14, and ACE 1. Sixteen months after the onset of treatment and over a year after its conclusion, Chris reported a BDI 0, Ham-A 2, and PCL 19.

#### Mental status examination

At his initial evaluation Chris' mother did most of the talking while he sat quietly and politely. Dress and grooming were appropriate. When we spoke to him alone, despite clearly attempting to be cooperative, he displayed poor eye contact. His speech was quiet, and responses were question-driven and minimal with mild speech latency. His mood was anxious, and his affect was constricted. The thought process was linear, and he expressed a desire to be able to “do more things without fear taking over,” as well as openness and optimism about the prospect of working together. Short-term and long-term memories were intact as evidenced by the ability to discuss recent and remote events and what brought him to treatment. There was no evidence of delusions or hallucinations, and he denied suicidal or homicidal ideations. Insight and judgment were fair.

At his first KAP session, Chris was administered a 100 mg rapid dissolve tablet, with a second 100 mg tablet given 22 min later. His experience was peaceful and playful, and he reported pleasant images and memories with a sense of his “brain relaxing.”

At his first intramuscular session, he disclosed a severe phobia of needles. Terrified and crying in anticipation, his father held his hand and gently encouraged him. After an hour of working through the fear with determination, Chris was able to receive the injection, resulting in a sense of accomplishment and a pleasant ketamine experience. In subsequent sessions, he received injections without significant difficulty.

The process of therapy unearthed an awareness of ongoing grief over his grandmother's recent death. He experienced his grandmother's presence during his sessions and felt that she wanted him to know he was “loved by everyone.” He gained an intuitive sense of meaningful actions he could take to process her loss; after an at-home session, he asked his mother to take him to her former house and in the yard, he encountered a swarm of dragonflies. He experienced this as a symbol of connection to her, engendering a sense of happiness, peace, and personal specialness.

Over the course of 5 months of treatment, lozenge dosage for at-home sessions ranged from 100 to 200 mg SL two times weekly. The intramuscular dose range was 50–70 mg, with a total of five in-office visits. There were no adverse medical events reported. Some experiences were described as “scary and difficult” but tolerable. The patient and his parents described continuing improvement in his social functioning; he resumed driving, attended large social events including a school dance, spent more time with friends, completed errands on his own, and went on a date. He began to speak more freely as our sessions progressed, stating “I feel like I have come back to life.” We terminated treatment in light of his improvement, leaving open the possibility of returning as needed.

Chris provides an example of KAP's utility for anxiety in a patient who had no prior experience with psychiatric medications, emphasizing the possibility for KAP as an effective short-term, intermittent treatment. While ketamine is often reserved for “treatment-resistant” patients, Chris's case suggests that postponing treatment with KAP may not be necessary or beneficial. Family involvement was essential to Chris' ability to participate in and trust the KAP process, as displayed in his session when facing and overcoming his phobia of needles. Finally, although it was not an explicit goal upon entering treatment, he came to recognize and process grief over the loss of his grandmother, with ketamine facilitating transpersonal experiences interacting with her thus fostering a sense of safety, connection, and belonging in the world.

#### Patient Chris' perspective comments

“One benefit to me after the ketamine experience is that I was able to accomplish many goals and tolerate situations I thought I would never be able to. Another benefit is that I don't[sic] feel as sad or depressed after my grandmother passed away, which put a very negative image in my head that I would think of every day until I got the ketamine therapy. I still get that image in my head today, but it doesn't[sic] make me sad anymore because I believe I have finally moved forward and accepted that she is gone.”

“The difficult effects from the medicine are that my whole body becomes numb and that for a while I forget where I am, what I am doing, and even forget who I am. It would sometimes get scary, and I would think that I was being sucked into a black hole and would be trapped in the journey forever. Another effect is when I wake up from the journey, it is hard to walk or even look straight and it takes a little over an hour for the medicine to leave my body after I eat something.”

“I will definitely do this again if something comes up in my life where I need to, and I would highly recommend it to troubled friends. Since I started it, I have recommended the psychotherapy to friends I know are going through hard times, and they even asked me questions of[sic] how it works and how it could help them.”

“I did not have trouble reducing and stopping the ketamine. The reason is that I felt ready to achieve the things I wouldn't have been able to achieve before.”

#### A comment from Chris's mother in follow-up over a year after conclusion of treatment

“He is driving now and thinking about what he's[sic] going to do after graduation. He has a better personality than before treatment. We are still working on certain things that he needs to get over like going through a drive-through and ordering food and eating out in a restaurant with his friends. He just needs a little push and once he does it and is successful, he is okay. He now gets gas for his car by himself. He is also working every weekend in the same position as before but now he is staying later; he's made friends with some of the prep cooks and stays to eat meals with the other employees. These are all things he would not do before treatment.”

### Patient Devon—Case example

keywords: bipolar disorder, eating disorder, major depression, ADHD, impulsivity.

Abstract: A now 23-year-old woman began treatment at age 19, with undiagnosed bipolar disorder and a complex history with multiple diagnoses, a history of impulsivity, and substance use, who was hospitalized abroad for a full manic episode early in our treatment of her, and who struggles with medication compliance. Her course of treatment has been erratic despite fluctuating use of valproate. KAP treatment has provided a decrease in the intensity and frequency of depressive episodes, a strong therapeutic bond, and intensive work with her parents has provided a degree of safety. Our perspective is that she will continue to fluctuate with symptoms and impulsivity and will continue to have difficulty sustaining a balanced life. The role of ketamine and KAP with Devon is to provide periods of relief from depression and impulsivity and support for her safety.

Devon is a now 23-year-old woman who began treatment with us when she was 19 years of age. Her treatment is ongoing as she has complex circumstances and comorbidities. Her initial diagnoses were a continuation of those from prior treatment and were validated at intake included major depression (F33.1), moderate to severe anxiety (F41.9), an eating disorder (ED) comprising both binging, purging, and restriction (F50.01 and F50.02), suicidal ideation of fluctuating severity (R45.851), OCD (F42), and ADHD—inattentive type (F90.9). Early in our relationship after a hospitalization abroad for mania with paranoia, her diagnosis was revised to include bipolar I disorder. In view of this, other diagnoses may reflect an earlier less symptomatic course of bipolar I disorder. Binge drinking and regular marijuana use occurred between the ages of 14 and 19. The mother's personal history suggests a bipolar I disorder, as does the maternal grandmother, which has consistently been denied them. The fact that the mother herself suffered from a bipolar illness was considered a culturally shameful idea and was not easily talked about. There are no other family, genetic, or mental illness issues of relevance. Her parents remain married, and she has one older brother.

#### Mental status evaluation

At intake, Devon is a 19-year-old Caucasian female who appears as her stated age; she is 5'3”, of normal stature, and appears as underweight. She presented with good eye contact, good hygiene, and grooming, and was casually dressed. Her affect at intake was normal and congruent with the topics we discussed. She appeared intelligent and seemed to grasp the impact of her symptoms. She demonstrated insight into problem areas of depression, and at times, impulsivity. She admitted to having difficulty in focus and concentration with her junior college schoolwork at times. She gave a fluent history of her difficulties as a teen. Her speech, while soft-spoken was of normal cadence. There is no history of seizures, periods of loss of consciousness, or any history of developmental delay. Her thought process was fluent with no signs of hallucinations or delusions. She admitted to frequent fleeting suicidal ideation off and on. This appeared to follow the deepening of depressive symptoms and shame. She has a long history of an eating disorder—binge and purge type, as well as restrictive type. She was hospitalized in the past for these disorders. She is very aware of her compulsion to restrict or binge/purge. This stems from the idea of being “too fat” while also acknowledging that she is very slender. She has some difficulty staying asleep and may get up at night when awake to eat, then purge.

Devon's baseline measures: BDI-38, PCL- 36, HAM-A −25, ACE – 3, RES- 8.

Measures at the time of this writing are as follows: BDI-24, PCL- 28, HAM -A−17. Her measures tend to fluctuate depending on her stability and swings from depression to hypomania.

#### Devon

Historically, there have been periods of extreme exercise aimed at achieving an idealized weight in conformity with body dysmorphia. Hospitalized multiple times for eating disorders at inpatient ED rehab centers, she had also attended partial outpatient programs. Her treatment team has included a nutritionist and a therapist focused on her ED. In terms of medications, she had been prescribed many different SSRIs, as well as atypical antipsychotics.

Following the hospitalization for mania, she has prescribed olanzapine, which was reduced during our care of her. She has great concerns about gaining weight and with the subsidence of her mania, olanzapine was reduced, and she came to use it for a time episodically for an increase in symptoms at a low dose. Her determination to use medication as she sees fit is a roadblock to a sustained balance. Olanzapine was discontinued by the patient almost 1 year ago after episodic use at 2.5–5 mg. Of interest is that Devon's weight remained stable despite the olanzapine which is known for its weight gain. Valproic acid ER was added with a dosage between 750 and 1,000 mg. Compliance with this has been an issue at times. Control of manic symptoms has been essential for our ability to utilize ketamine, which has no effect on mania. As she became pregnant at one point, then miscarried, given valproates teratogenicity and erratic use of birth control, a decision to substitute with a trial of lamotrigine was initiated recently with Devon stopping its use at low dose before any possibility of drug effect could occur.

Devon is attractive and somewhat shy, a gifted artist with a striking portfolio of paintings and drawings. She is 5'3” and has weighed between 102 and 110 lbs. Devon has difficulties with focus and concentration, and as a result, has not been able to follow a full school program. However, recently she finished three semesters at the local community college. Recently has dropped out in a depressive episode triggered by an eruption of acne that caused her to feel self-conscious in public.

Devon was referred to us by her parents, who had grown worried and frustrated by her lack of symptom control, the inadequacy of medication management, and confusion about her diagnosis. In addition, Devon and her parents were concerned by her fluctuating symptoms of hypomania, depression, anxiety, difficulty concentrating, inability to follow a consistent path of development, impulsivity, and eating disorder. Her case represents the significant difficulty of treating adolescents with complex disorders and a long history of failed psychiatric and psychotherapeutic interventions.

Previous treatments beginning at age 14 included multiple SSRIs (TRD by definition), atypical antipsychotics, treatment at multiple eating disorder rehabs and with eating disorder specialists, psychotherapy, and behavioral health treatment centers—both inpatient and IOPs.

Devon, a gymnast in elementary and middle schools, had a body shaming incident at the age of 11 that seemed to trigger her anxiety and obsession with her weight and body image. There was also an inappropriate sexual advance from a guidance counselor at age 14 in high school. After that, she began drinking, partying, and having indiscriminate sex, and her eating disorder became prominent.

Excelling in athletics was important in her family. While supportive and concerned, her mother was wary of medications and has continued to emphasize healthy living and will-power as the solution to Devon's problems, complicating treatment and compliance. The maternal family history suggesting bipolar I disorder only came to light later in treatment.

Four months after beginning treatment with KAP, Devon went on a trip abroad to see her mother, brother, and extended family. During this trip, she experienced a full manic episode that required hospitalization. This appears to have been precipitated by the 24 h of daylight during the Scandinavian summer, jet lag, and resulting five nights of sleep deprivation. This break moved us to treat her as having bipolar I disorder, and we came to recognize that her behavior in high school was consistent with this. Age at onset of bipolar I disorder (BPD) typically begins at 12–24 years of age. An earlier presentation of BPD-I may lead to a more severe course (39).

#### Course

The complexities of her history and emotional and family life led to a multifactorial view of causation and a multipronged treatment approach.

Ketamine treatment followed the relative stabilization of her manic symptoms and proceeded according to our protocol, W began with our SL dosage escalation to assess sensitivity to the medicine. With this established, we developed a closely supervised SL protocol for at-home sessions to further control symptoms of depression, anxiety, and reduce suicidal ideation. In-office sessions moved to IM ketamine administration for a more profound release from her usual concerns and obsessions. Ketamine is usually administered to her in divided doses to sustain the duration of its effect because of her rapid metabolism. Prolonging the duration of ketamine's effect in terms of additional minutes improves the effect of the experience and relief from symptoms.

Devon's course has alternated between periods of relative stability and increased social and personal growth, with periods of severe depression, intense purging, anxiety, and hypomanic symptoms. Both the olanzapine and valproate have been of benefit when used regularly, particularly the valproate which has reduced hypomanic symptoms and prevented another full manic episode. Devon is not alone in her manner of dealing with medication. Adolescents we have treated, often have tended to resist taking medications fearing their effects and at times projecting a lifetime of use. Many have also failed other psychiatric medications and are wary of side effects, having experienced their unpleasantness. In contrast, alcohol, marijuana, and other drugs may be consumed without such concerns despite the problems experienced by their use. In Devon's case, she has continued to use cannabis for anxiety, sleep, and its effects. She also drinks wine at times with family and friends. The former has led to some impulsive acting out that further complicates her life. She has not discontinued use despite our recommendations and the consequences.

While Devon was wary of other medications, she preferred ketamine because of the absence of side effects, the nature of the experience, and because it is not a daily medication. KAP has helped with depression but has not eliminated all episodes. Some of which are sudden and precipitous; conducting KAP sessions when these decompensations occur has reduced their duration.

In another ketamine format, we have implemented a preventative protocol of low-dose ketamine sessions at home to assist with her eating disorder. We are attempting to engage her in blocking self-destructive impulses. When the urge begins to engage in eating disorder behaviors (binging, purging, restricting), Devon is encouraged to put the impulse aside with the relaxing effect of a 50 mg ketamine lozenge. Choosing this action is one of creating awareness and taking agency to diminish the anxiety that precedes binging and purging allaying the impulse based on ketamine's anxiolytic property. This interruption of her pattern combined with higher dose ketamine sessions once or two times per month now has succeeded in eliminating binging and purging episodes.

In-office ketamine IM sessions have resulted in the lessening of depression—shorter and less deep episodes. Her suicidal ideation has markedly decreased and is often not present. Periods of happiness and constructive activity have become more frequent. IM ketamine sessions have been well-tolerated and often enjoyed. Devon has never been agitated or fearful, but rather quite peaceful during and after IM sessions. Her sense of a time-out from her ordinary way of thinking replaced by an experience of traveling and moving through space freely and imaginatively has engendered important changes in her view of herself affecting her identity formation positively.

To date, Devon has had 38 ketamine sessions in-office and many more at home.

Despite setbacks, what has been apparent over the course of these almost 2 years is a substantial increase in insight, clarity, maturity, and self-awareness. Her parents who are involved in her treatment have said “This saved her life.” She has now completed three semesters at the local junior college and maintained over a 3.0 average. She has volunteered at an art studio to teach children art and has sold a few of her own pieces. She was involved in two romantic relationships in the past year and ended both when she realized they were unhealthy. One led to pregnancy and miscarriage.

The partial success of treatment with this complex young woman results from several factors: her trusting relationship with her therapist; her honesty with the psychiatrist; the flexibility and advocacy we have shown with her medications; the initiation of mood stabilizers to control her mood swings; her consistency with ketamine and therapy; maturation and containment; insight and agency in handling emotional lability; developing and maintaining the support of her family; and her growth in self-confidence and self-worth. To manage at this young age the constant movement of her center due to bipolar I disorder is truly difficult and the impulsive actions that result from it dislocate her trust in herself and her judgment and necessitate periods for recovery and reorientation. Lack of medication compliance frustrates treatment efforts and makes medication remediation difficult.

We present Devon as an example of the mitigation of a severe mental disorder by ketamine as an assisted psychotherapy. Not its complete remission. Ketamine in such circumstances is not a stand-alone medicine, rather requires a full psychiatric and family therapy treatment approach. As there are so many people who face these sorts of difficult lives, and so many are young when interventions may have more effect, we advocate for exploring KAP's use with this population on a person-by-person basis.

#### Patient Devon's perspective comments

A few years ago, I was severely depressed. I felt like there was no hope for the future and I would be doing the world a service by committing suicide. I felt so disgusted with myself [sic]all I wanted to do was hide. I'd[sic] tried various medications: Abilify, Celexa, Prozac, Lamictal, Buspar, Geodon—the list goes on. I grew up a very happy child, but I developed an eating disorder around the age of 12 that robbed me of my wellbeing and sanity. Along with this came long depressions[sic] as well as something, I felt was a bit like mania, I would become very obsessive and was anxious a majority of the time. I didn't not[sic] feel I could trust or control my mind at all. When I was depressed, all I could think about was suicide, I spent years in and out of treatment centers and mental hospitals. The last few years, I've[sic] done better with eating, but it is still a struggle. A few years ago, I got diagnosed with bipolar disorder after a manic episode that landed me in the hospital. Since then, I've[sic] stayed out of the hospital, I think largely thanks to my therapist's support as well as the support from my family and friends. I'd [sic]never thought even a year ago that I would be able to say that I would be able to go 6 months without purging. I feel ketamine has really helped tremendously with my suicidal thinking as well as obsessive thinking and anxiety around food and my body. I feel after a ketamine experience, especially after I am very depressed, I can see life and myself in a new light. I am able to focus more on the good things in life, be more compassionate toward myself and others, and the little voice in my head telling me I'm not good enough seems to fade into the background.

Using the low dose[sic] ketamine, I tend to not crave alcohol, and I have been able to cut down my cigarettes to 1 or 2 a day.

The journey itself can be a beautiful and comforting experience or it can be terrifying. There was one experience when I felt I was on my deathbed, I became extremely panicked trying to find a way to live, [sic]I guess I screamed out “I don't want to die”. After this session, I felt like a new person. I was so much more positive and confident in my abilities. I felt that my fears didn't[sic] control me anymore. I have been preoccupied with suicide since I was 12 but I feel my experiences with ketamine have changed something in my brain almost and now I don't[sic] obsess over the idea. I feel that the treatments have helped tremendously with my eating disorder also.

I feel ketamine does help with depression, anxiety, and my obsessive thinking so I notice when I don't[sic] take the medication I have a harder time, but it's[sic] one of the only drugs I think I've[sic] taken that doesn't[sic] have withdrawals so when I stop using it I don't[sic] have a physical craving for it, but I would say I do have a mental craving for it at times because it does help me a lot. I notice I will go to alcohol more often when I'm[sic] not using ketamine to calm my anxiety around food at times.

## Discussion

We have presented four case examples of the treatment of adolescents with a wide array of emotional disorders utilizing family-centered KAP methodologies. We have been treating adolescents for over 3 years and there are many more cases we could discuss which resulted in the remission of symptoms and emotional maturation. There are also those with partial successes and failures. Providing therapy to adolescents with significant emotional difficulties tends to be a complex and often arduous undertaking with many variables affecting the course of treatment and its outcome (61).

The ketamine experience itself tends to be the least of the difficulties faced, though we have lost several patients to intractable nausea and vomiting despite our best efforts to ameliorate these symptoms. This is the most common SE occurring in about 5% of our patients overall (18). Ketamine is not for everyone. However, with that caveat, once comfortable with the disorientation and the reduction of contact with this reality and even its transcendence, tolerance and embracing of the ketamine state occur, often with relief and new organization of character and behavior on return and through the support for integration and change by the psychotherapists.

In fact, our adolescent population tends to cope well with the altered states of ketamine. Even those who have significant experience with prior substance use differentiate ketamine experience. We have had no incidents of patient drug-seeking for ketamine outside of our clinical practice. With the essential requirement of close parental control of the SL lozenges we prescribe for at-home use, there has been no diversion to friends or others. Often, our patients have reported that their peers have recognized their positive changes and expressed interest in experiencing therapy.

We are a full-service psychiatric provider, and screening patients for appropriateness prior to initiating KAP treatment is essential. In our experience, factors that may diminish the effectiveness of KAP include recurrent psychotic episodes, a history of untreated/partially treated hypomania, a tendency toward impulsivity and acting out, medication nonadherence, and substance use (18). While we reported on this in our article on adults with 235 patients, hundreds of additional patients and thousands of KAP sessions have continued to provide the same information and will be reported in a forthcoming update of our clinical practice. In this adolescent population, the same factors appear to be impactful.

The formal search for moderators of the effect of ketamine treatment has not yet revealed consistent patient-level clinical or demographic features to help guide the precision application of ketamine (62). Furthermore, while a recent meta-analysis (63) did not detect a difference in response to IV ketamine in bipolar vs. unipolar depression patients, our clinical experience in our particular format for the application of ketamine has suggested that a history of untreated or partially treated bipolar disorders diminishes the effectiveness of KAP. Unstable, stressful, and conflictual family situations are also influential on adolescent emotional integrity and often causative of dysregulated behavior. Economic factors, peer group loyalties, unstable love relationships, a history of abuse, and the presence of perpetrators with a lack of parental protection are all factors and must be addressed along with the actual treatment. Families and parents are often highly stressed by their difficulties in coping with their adolescent(s). Divorce and splitting between parents are among adverse childhood experiences with a significant impact on adolescent mental health and emotional integrity.

In short, the myriad familial, social, and cultural contexts—racism, gender discrimination, and misogyny among them—impact our ability to facilitate autonomy, individuation, and introjection of self-control and self-interest.

In light of the above, administering ketamine as a drug without a strong psychotherapeutic presence is likely not a recipe for success. Engagement in the totality of an adolescent and their situation is essential to the development of trust in the therapist and the success of treatment. Adolescent identity development is predicated on a growing sense of self that is stabilized and leads to commitments to constructive paths. Disruption of this process leads to confused and unstable identity formation (64), and if carried through into adulthood this disintegrated identity leads to poorer or better life integrations and social and physical health (65).

Our work is beginning. Larger numbers and data on how factors including diagnosis and personality characteristics affect treatment will lead to better treatment and patient selection. Reliability in attending treatment sessions and parental involvement in-office and at home is essential to the careful use of this medicine. Mathai's hopeful survey of parental attitudes (49) indicates the desperation for help with their children. Supporting adolescent needs for respect for their growing independence, and autonomy is imperative for guiding the therapeutic work in the family crucible. We hope awareness of KAP will encourage parents to view this treatment not as the last option prior to giving up or hospitalization, but rather as an early therapeutic opportunity to grow families closer, aiding adolescents in healing from the pain and trauma that has marked their lives.

We expect that this work will generate controversy as “drugs” and children are difficult subjects and there will be concerns raised about the effects on growing minds and bodies, on KAP being a gateway to other substance use, and the ability of an adolescent to handle the potentially disorienting effects of ketamine. These are legitimate concerns. Initial clinical evidence and our experience are reassuring. Uncontrolled adolescent drug use is rampant and can be harmful on the experiential level with detrimental sets and settings (66). We make a point of providing education to our young population not only about ketamine but also about other psychedelics. We teach and inform them about the safe practice at home with ketamine regarding set, setting, safety, dosage, having an alert, and a safe person nearby. We do not promote the use of psychedelics in young people, but we do extrapolate the knowledge of ketamine to other psychedelics-teaching them harm reduction principles should they or their friends at some point in time choose to use psychedelics. Our younger population appreciates this and has given advice to friends and family. They learn to respect the power and intentionality of these medicines.

From NCDAS for 2019 helping us to grasp the situation in the US—a few of their probably low estimate statistics:

- 591,000 teenagers aged 12–17 years old used an illicit drug other than marijuana in the last month.
- 8.7% of eighth graders have used illicit drugs in the last month.
- 21.3% of eighth graders have tried illicit drugs at least once.

By the time they are in 12th grade, 46.6% of teens have tried illicit drugs.

Our KAP process opens the door to adolescents and their families for a frank discussion of drug use, unlike virtually any other setting. Many of our adolescents are drug-naive but drug aware and, in their minds, and social groups, it is looming ahead. Others have been too experienced for their own good. The KAP experience provides a model for a safe and supported set and setting, the essential factors in any drug experience [67]. Teaching it as sacred and a journey into the depths of the mind focuses away from “recreational” use and drug culture, toward conscious thoughtful experience. KAP differentiates “medicine” from “drug.” Enabling an honest and open path between parents and their children leads to a non-oppositional stance and an ability to ask for parental support and discussion in advance of drug use. KAP for adolescents provides us with the opportunity to educate, discuss, and exemplify safe use and harm reduction, encouraging postponement of substance use to a time when the brain and mind have more capacity to handle their effects. On the experiential level, it enables our adolescents to experience themselves deeply and to reflect on their lives and choices.

## Conclusion

Ketamine-assisted psychotherapy is a new approach to the family-centered adolescent treatment of a variety of mental disorders and acting out states that are afflictive and unique to those in this age range. It offers an opportunity for rapid resolution of suicidality as well. Careful preparation of patients and family, coordination of care, and intensive psychotherapy are component parts of the KAP process. Ketamine itself offers a time-limited respite from the ordinary mind that varies in intensity with dosage and personal sensitivity. This enables relief from obsessional states and preoccupation allowing for a new approach to life through a new experience of self. Ketamine serves as a front-line antidepressant that is administered intermittently, is safe with over a 50-year history of use, and is compatible with most other psychiatric medications given its different neurotransmitter modes of action. We have presented four case examples at length to provide a preliminary sense of our modality.

## Data availability statement

The original contributions presented in the study are included in the article. Further inquiries can be directed to the corresponding author.

## Ethics statement

Ethical review and approval was not required for the study on human participants in accordance with the local legislation and institutional requirements. Written informed consent to participate in this study was provided by the participants' legal guardian/next of kin. Written informed consent was obtained from the individual(s) AND/OR minor(s)' legal guardian/next of kin for the publication of any potentially identifiable images or data included in this article.

## Author contributions

All authors have participated in contributing to the article through their clinical work and writing or the paper including case examples and editing. All authors contributed to the article and approved the submitted version.

## Footnotes

^1^Assessments: ACE - adverse childhood experiences; Res - resilience; BDI - Beck Depression Inventory; HAM-A - Hamilton anxiety scale; PCL-C - PTSD checklist/civilian; MEQ - Mystical Experiences Questionnaire; EDI - Ego-Dissolution Inventory/Short.
